# D-Limonene Alleviates Acute Kidney Injury Following Gentamicin Administration in Rats: Role of NF-*κ*B Pathway, Mitochondrial Apoptosis, Oxidative Stress, and PCNA

**DOI:** 10.1155/2021/6670007

**Published:** 2021-01-13

**Authors:** Esmaeel Babaeenezhad, Forouzan Hadipour Moradi, Sobhan Rahimi Monfared, Mohammad Davood Fattahi, Maryam Nasri, Abdolhakim Amini, Omid Dezfoulian, Hassan Ahmadvand

**Affiliations:** ^1^Department of Clinical Biochemistry, School of Medicine, Student Research Committee, Shahid Beheshti University of Medical Sciences, Tehran, Iran; ^2^Razi Herbal Medicines Research Center, Faculty of Medicine, Lorestan University of Medical Sciences, Khorramabad, Iran; ^3^Department of Clinical Biochemistry, Faculty of Medicine, Lorestan University of Medical Sciences, Khorramabad, Iran; ^4^Department of Pathobiology, School of Veterinary Medicine, Lorestan University, P.O. Box 465, Khorramabad, Iran

## Abstract

Clinical application of gentamicin (GM) is well known to be associated with the development of acute kidney injury (AKI). This study was the first to investigate the possible protective effects of D-limonene (D-lim) on AKI following GM administration in rats. 32 rats arranged in four groups (*n* = 8): (1) the control group received saline intraperitoneally (0.5 ml/day) and orally (0.5 ml/day), (2) the D-lim group received D-lim (100 mg/kg) orally and saline (0.5 ml/day) intraperitoneally, (3) the GM group received GM (100 mg/kg/day) intraperitoneally and saline (0.5 ml/day) orally, and (4) the treated group received intraperitoneal GM (100 mg/kg) and oral D-lim (100 mg/kg). All treatments were performed daily for 12 consecutive days. Results revealed that D-lim ameliorated GM-induced AKI, oxidative stress, mitochondrial apoptosis, and inflammation. D-lim showed nephroprotective effects as reflected by the decrease in serum urea and creatinine and improvement of renal histopathological changes. D-lim alleviated GM-induced oxidative stress by increasing the activities of renal catalase, serum and renal glutathione peroxidase, and renal superoxide dismutase and decreasing renal malondialdehyde and serum nitric oxide levels. Intriguingly, D-lim suppressed mitochondrial apoptosis by considerably downregulating Bax and caspase-3 (Casp-3) mRNA and protein expressions and markedly enhancing Bcl2 mRNA and protein expressions. Furthermore, D-lim significantly decreases GM-induced inflammatory response through downregulation of NF-*κ*B, IL-6, and TNF-*α* mRNA and/or protein expressions and decrease in renal myeloperoxidase activity. Finally, D-lim remarkably downregulated PCNA protein expression in the treated group compared with the GM group. In brief, this study showed that D-lim alleviated AKI following GM administration in rats, partially through its antioxidant, anti-inflammatory, and antiapoptotic activities as well as downregulation of PCNA expression.

## 1. Introduction

Gentamicin (GM) is an aminoglycoside antibiotic, which is clinically beneficial against infections induced by Gram-negative bacteria. In spite of GM's valuable role in treating various bacterial infections, its clinical application induces acute kidney injury (AKI), and therefore, the utilization is restricted in clinical trials. It is well known that GM causes dose-dependent nephrotoxicity [1–4], which is related to its accumulation in the renal proximal convoluted tubules. Nephrotoxicity is distinguished by functional and morphological changes in the kidney such as elevated blood urea nitrogen and creatinine (Cr) in serum, declined glomerular filtration rate (GFR), edema, and acute injury in proximal tubules [5, 6]. The underlying mechanisms involved in the pathogenesis of GM-induced AKI are still not well known. However, it has been suggested that different factors such as mitochondrial dysfunction, oxidative stress, NF-*κ*B activation, inflammation, apoptosis, necrosis, phospholipidosis, and inducible nitric oxide synthase (iNOS) activation are involved in the pathogenesis of GM-induced AKI [7].

Several studies showed that GM amplifies the generation of reactive oxygen species (ROS) in the kidney. ROS exert cytotoxic effects through lipid peroxidation, protein oxidation, and apoptosis induction. Additionally, ROS have a main role in the progression of inflammation via NF-*κ*B activation [8]. Enhanced ROS generation followed by GM administration produces free NF-*κ*B, which translocates to the nucleus and stimulates the target genes involved in the inflammatory process, including inducible nitric oxide synthase (iNOS), cytokines (TNF*α* and IL6), and adhesion molecules. These downstream genes contribute to the pathogenesis of GM-induced AKI [9–11].

The elevation of ROS during GM-induced AKI has a principal role in the activation of the intrinsic pathway of apoptosis through mitochondrial dysfunction [12]. In this route, Bax and Bcl-2 as mitochondrial-associated proteins are the major determinative molecules, in which maintenance of the ratio in Bcl-2 to Bax prevents apoptosis; however, imbalance in this ratio terminates to increase permeability of mitochondria and release of cytochrome c into the cytosol, which in turn activates caspase-9 (Casp-9) and caspase-3 (Casp-3). The activation of these enzymes finally leads to DNA fragmentation and cell death [13, 14].

Proliferating cell nuclear antigen (PCNA) is a p53-dependent molecule that plays two different roles including DNA replication, when p53 is decreased, and DNA repair, when p53 is increased [15]. P21 is another p53-dependent protein and interacts with PCNA in order to arrest the cell cycle following the potential role of PCNA in DNA repair [15–17]. Previous studies reported the upregulation of PCNA, P21, and P53 in renal cells in response to intoxication with GM [18–20].

**Figure 1:**
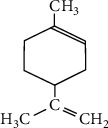
Chemical structure of D-limonene (D-lim) [32].

D-Limonene (D-lim), also known as 1-methyl-4-(1-methylethenyl) cyclohexene (Figure 1), is a monocyclic monoterpene commonly found in orange and many citrus oils [21]. It constitutes more than 95% of terpenoid compounds in ripe oranges [22]. Different properties of D-lim such as anticancer [23], antioxidant, anti-inflammatory [24], antiapoptotic [25], and antidiabetic [26] effects have been reported in published data. D-Limonene prevents mitochondrial dysfunction, scavenges ROS, and protects cells from oxidative injuries [25, 27]. Moreover, other biological characteristics such as hypolipidemic and immunomodulatory activities and hepatoprotective, dermatoprotective, and chemopreventive effects are associated with D-lim [21, 28, 29]. Additionally, it has been demonstrated that D-lim plays a main role in the improvement of the gallstone and doxorubicin-induced renal injuries [30, 31].

To the best of our knowledge, D-lim effects on GM-induced AKI have not yet been investigated. Therefore, this study was designed to determine potential protective effects of D-lim on GM-induced AKI. We investigated the effects of D-lim on oxidative stress and inflammatory biomarkers, mitochondrial apoptosis, PCNA expression, and histopathological alterations in a rat model of GM-induced AKI.

## 2. Materials and Methods

### 2.1. Chemicals

Tris-ethylenediaminetetraacetic acid (Tris-EDTA) and Tris-HCl were provided from Merck Company (Germany). 2,4-Dinitro thiocyanate benzene (DNTB), K_2_HPO_4_, H_2_O_2_, NaN_3_, D-lim (≥95.0%), GM, pyrogallol, and glutathione (GSH) were purchased from Sigma-Aldrich Company (USA). Commercial kits for the measurement of urea and Cr were purchased from Pars Azmoon Company (Iran). A complementary DNA (cDNA) synthesis kit and SYBER Green qPCR Master Mix 2x were taken from Yekta Tajhiz Azma (YTA) Company (Iran).

### 2.2. Animals and Experimental Design

In this study, thirty-four adult male Wistar rats (mean weight: 180 ± 20 g) were taken from the Pasteur Institute (Tehran, Iran) and transferred to the animal maintenance department of Razi Herbal Medicines Research Center (Lorestan, Iran). The rats were kept in a room with controlled conditions for laboratory animals, including temperature of 23 ± 2°C, humidity of 50%, 12 h light/dark cycles, and free access to food and water. These animals were housed in a room 7 days before the start of the study in order to comply with the above conditions. The animals were randomly divided into 4 groups (8 rats per group):

Control group: saline was intraperitoneally (0.5 ml/day) and orally (0.5 ml/day) administrated for 12 consecutive days.

D-lim group: D-lim (100 mg/kg) [32] was given orally, and saline (0.5 ml/day) was administrated intraperitoneally for 12 consecutive days.

GM group: animals received daily GM (100 mg/kg, intraperitoneally) [33, 34] and saline (0.5 ml/day, orally) for 12 consecutive days.

Treated group (GM+D-lim): GM (100 mg/kg) was given intraperitoneally, and D-lim (100 mg/kg) was administrated orally for 12 consecutive days.

D-lim was administrated to animals 1 hour after intraperitoneal GM injection [35]. The dose (100 mg/kg) was selected according to a previous study representing the therapeutic effects of D-lim on streptozotocin-induced diabetes mellitus in rats [32]. Oral administrations were performed using gavage. Saline was used as a vehicle for D-lim.

All the experiments conducted in this study, as well as animal work protocols, were in accordance with the methods approved by the university's Ethics Committee and the US National Institutes of Health (NIH 1978). Moreover, the Animal Ethics Committee of Lorestan University of Medical Sciences approved our experimental protocols.

### 2.3. Sample Collection

After 12 days, the animals in all groups were anesthetized with ketamine (87 mg/kg, intraperitoneally) and xylazine (13 mg/kg, intraperitoneally) [36, 37]. The blood sample was taken from the heart of each animal using a syringe, and then serum was isolated by centrifuging at 4°C (15 minutes, 3000 rpm). Serum samples were kept at -20°C for biochemical evaluations. The left kidney was dissected into two equal parts. One section was homogenized with 10% PBS buffer for renal biochemical assessments, and the other was kept at -70°C for the evaluation of gene expressions. The right kidney was stored in neutral buffered formalin (10%) for histopathological and immunohistochemical evaluations.

### 2.4. Biochemical Analysis

#### 2.4.1. The Measurement of Renal and Liver Functional Markers

The levels of urea and Cr in serum were measured by using a biochemical autoanalyzer (Olympus AU-600, Tokyo, Japan) applying commercial kits (Pars Azmoon, Tehran, Iran).

#### 2.4.2. The Evaluation of Oxidative Stress and Inflammatory Biomarkers

The concentrations of malondialdehyde (MDA), as the marker of lipid peroxidation, in the serum and kidney of the rats were measured based on the thiobarbituric acid (TBA) assay [38, 39], which was fully described in our previous study [40]. The serum and renal levels of GSH were determined spectrophotometrically at 412 nm according to Ellman's method [41]. Nitric oxide (NO) levels in the serum of rats were evaluated by the determination of nitrite as the end product of NO. For the determination of nitrite level, the method of Giustarini et al. was used in this study [42]. The serum and kidney activities of catalase (CAT) in all groups were assayed by Sinha's method [43]. Glutathione peroxidase (GPX) activities were evaluated according to Rotruck et al.'s method [44]. The assessment of superoxide dismutase (SOD) activity was performed spectrophotometrically (420 nm) based on the prevention of interaction between superoxide radical and pyrogallol [45]. The evaluations of myeloperoxidase (MPO) activities in renal tissue were carried out based on hydrogen peroxide-caused O-dianisidine dihydrochloride oxidation at 450 nm [46].

#### 2.4.3. RNA Isolation and Quantitative Real-Time RT-PCR (QRT-PCR)

Total RNA was isolated from the kidney samples using the TRIzol reagent (MO, USA) as fully described in our previous study [47], and RNA was kept at -80°C until utilization. The quantity and purity of isolated RNA were evaluated by using a NanoDrop spectrophotometer (Biochrom WPA Biowave II, UK), and samples with 260/280 and 260/230 nm ratios of about 2 were selected. RNA integrity was determined by electrophoresis (2% agarose gel). After that, cDNA was synthetized from total RNA samples (2 *μ*g) using the cDNA synthesis kit (YT4500, Yekta Tajhiz Azma, Iran) based on its manufacturer's protocols. Then, the expression levels of target genes (TNF-*α*, IL-6, NF-*κ*B, Bax, Bcl2, and Casp-3) and the reference gene (GAPDH) were assessed by quantitative real-time reverse transcription polymerase chain reaction (QRT-PCR) using SYBER Green qPCR Master Mix 2x (YT2551, Yekta Tajhiz Azma, Iran). All reactions were performed in triplicate on Rotor-Gene 6000 (Corbett Research) under the following thermal cycling condition: 1 cycle of 95°C for 3 min and 40 cycles of 95°C for 5 sec (denaturation) and 40 cycles of 60°C for 20 sec (annealing and extension). The 2^−*ΔΔ*CT^ method was used for the calculation of relative mRNA expression [48]. The sequence of utilized primers is listed in Table 1.

### 2.5. Histopathological Evaluation

Tissue samples were fixed in 10% neutral buffered formalin and following dehydration in ascending grades of ethyl alcohol were cleared in xylene and embedded in paraffin. Paraffin sections of kidneys were cut at 5 *μ*m on a rotary microtome, mounted on slides, and stained with hematoxylin and eosin (H&E).

Acute tubular necrosis (ATN) of the epithelium was assessed in full thickness of the cortex, which was divided into three distinct areas, including subcapsular region (supracortex), cortex, and corticomedullary junction (subcortex) which were scored according to the degree of epithelial damage using a zero through five grading system: zero, no lesion; one, ≤10% ATN; two, 11% to 25% ATN; three, 26% to 50% ATN; four, 51% to 75% ATN; and five, 76% to 100% ATN [49].

### 2.6. Immunohistochemistry (IHC)

3 *μ*m dewaxed and rehydrated renal tissues were immersed in target retrieval solution (Tris-EDTA, pH 9.0) and boiled in a water bath for 20 min at 98°C to deliver unmasked antigens. Then, the sections were treated with 3% H_2_O_2_ in PBS for 15 min to block endogenous peroxidase, and nonspecific background staining was blocked by incubating the sections with 5% normal rabbit sera in PBS for 20 min. Sections were incubated 1 h with polyclonal rabbit anti-Casp-3 (orb 10237, Biorbyt, UK) at 1 : 50 dilution, PCNA (orb 128497, Biorbyt, UK) at 1 : 50 dilution, Bcl-2 (orb 10173, Biorbyt, UK), TNF-*α* (ab6671, Abcam, UK) at 1 : 100 dilution, and Bax (sc 7480, Santa Cruz, USA) at 1 : 50 dilution and then incubated for 20 min with biotinylated goat anti-rabbit IgG (prediluted, Biocare, USA), followed by incubation with streptavidin horseradish peroxidase (sHRP) (prediluted, Biocare, USA) for 20 min. The antibody binding sites were visualized through reaction with DAB solution. Finally, sections were counterstained with Mayer's Hematoxylin (Bio Optica, Italy). Manual cell counting using an ocular micrometer (graticule) was applied for the % positive tubular cell IHC score by two pathologists independently.

Each slide was placed on the optical photomicroscope (Olympus CX31; Olympus, Philippines) and observed under the 10 objective for every marker other than PCNA. Four equal quarters were obtained in each field. In any quarter, the mean of 60 tubules was considered and positive stained tubules were counted (240 tubules/field). 30 fields randomly were selected for each slide. For PCNA, a 40 objective lens was used with 12 tubules/quarter (48 tubules/field) and 60 fields randomly were chosen [50].

### 2.7. Statistical Analysis

The Statistical Package for the Social Sciences (SPSS, version 18; SPSS Inc., Chicago, Illinois, USA) was used for analysis of data. All results were represented as mean ± standard error (SE). Distributions of data in each experimental group were determined using the one-sample Kolmogorov-Smirnov test. Normal distribution data were compared between groups using one-way ANOVA followed by a post hoc LSD test. Data with nonnormal distribution were compared between groups by the Mann–Whitney *U* test. A *P* value lower than 0.05 was accepted as statistically significant. All graphs were structured using GraphPad Prism version 8.0.2. (GraphPad Software Inc., USA) for Windows.

## 3. Results

### 3.1. Biochemical Analysis

#### 3.1.1. The Effect of D-lim on Renal Function Markers

**Figure 2:**
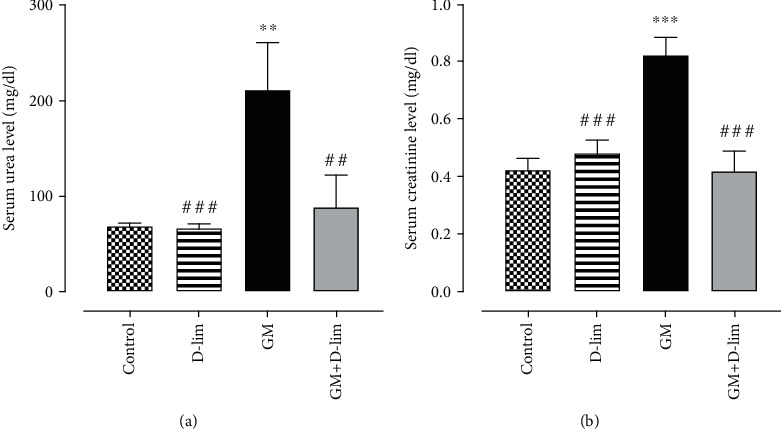
The effects of D-lim on the serum levels of renal function parameters including urea (a) and creatinine (b) in rats intoxicated with GM. Bars indicate 8. Data with normal distribution (creatinine) were compared between groups using one-way ANOVA followed by a post hoc LSD test. Nonnormal distribution data (urea) were compared between groups using the Mann–Whitney *U* test. ^∗∗^*P* ≤ 0.01 and ^∗∗∗^*P* ≤ 0.001, against the control group. ^##^*P* ≤ 0.01 and ^###^*P* ≤ 0.001, against the GM group.

As indicated in Figures 2(a) and 2(b), GM administration for 12 days caused a significant (3.07-fold and 1.95-fold, respectively) increase in the serum levels of urea and Cr compared with the control group (*P* ≤ 0.01). The administration of D-lim significantly (57.81% and 49.36%, respectively) inhibited the increment of serum urea and Cr in the treated group compared with the GM group (*P* ≤ 0.01), and their levels were similar to the normal levels observed in the control group (*P* > 0.05).

#### 3.1.2. The Effect of D-lim on Oxidative Stress and Inflammatory Biomarkers

**Figure 3:**
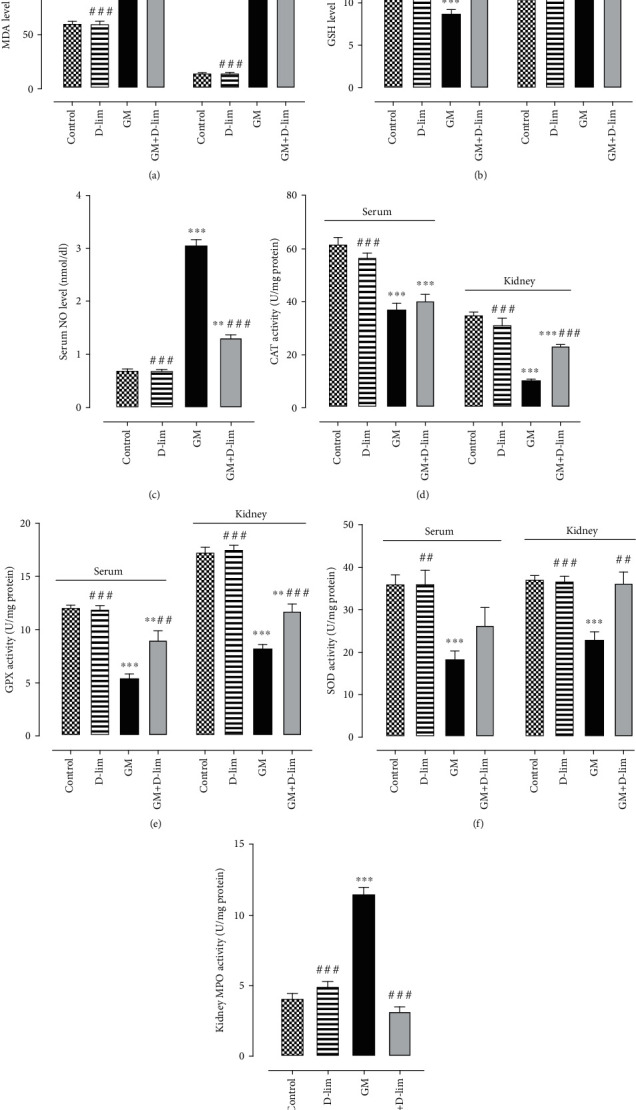
The effects of D-lim on serum and renal oxidative stress biomarkers in rats with GM-induced nephrotoxicity. Bars indicate mean ± SEM. Data with normal distribution (CAT and GSH) were compared between groups using one-way ANOVA followed by a post hoc LSD test. Nonnormal distribution data (MDA, NO, GPX, SOD, and MPO) were compared between groups using the Mann–Whitney *U* test. ^∗∗^*P* ≤ 0.01 and ^∗∗∗^*P* ≤ 0.001, against the control group. ^#^*P* < 0.05, ^##^*P* ≤ 0.01, and ^###^*P* ≤ 0.001, against the GM group.

The status of oxidative stress biomarkers is shown in Figure 3. Serum and renal MDA concentrations were significantly (1.85-fold and 12.58-fold, respectively) higher in the GM group than in the control group (*P* ≤ 0.001). In contrast, serum and renal GSH concentrations were significantly (1.79-fold and 1.59-fold, respectively) lower in the GM group than in the control group (*P* ≤ 0.001). The treatment of nephrotoxic animals with D-lim resulted in a significant (28.72%) decline in renal MDA concentration (*P* ≤ 0.001) and a significant (51.70% and 34.16%, respectively) elevation in serum and renal GSH concentrations as compared with the GM group (*P* ≤ 0.01). Furthermore, serum MDA level reduced (6.25%) in nephrotoxic rats following D-lim treatment, but it was not statistically significant (*P* > 0.05). Serum GSH concentration in the treated group was similar to the normal level seen in the control group (*P* > 0.05).

GM-injected rats showed a significant diminution in the serum and renal activities of CAT (1.65-fold and 3.34-fold, respectively), GPX (2.20-fold and 2.08-fold, respectively), and SOD (1.97-fold and 1.61-fold, respectively) in comparison to the control group (*P* ≤ 0.001). D-lim significantly promoted renal activity of CAT (123.74%), serum and renal activities of GPX (64.95%, and 41.68%, respectively), and renal activity of SOD (58.21%) in the treated group compared with untreated nephrotoxic animals (*P* ≤ 0.01). Additionally, D-lim remarkably enhanced serum activities of CAT and SOD (8.14% and 43.88%, respectively) in the treated group in comparison to the GM group, but these changes were not statistically significant (*P* > 0.05).

The serum level of NO and the renal activity of MPO significantly (4.34-fold and 2.80-fold, respectively) augmented following GM injection in nephrotoxic rats in comparison to healthy animals (*P* ≤ 0.001). Treatment of GM-intoxicated animals with D-lim significantly (57.06% and 72.18%, respectively) diminished serum NO level and renal MPO activity in the treated group compared with the GM group (*P* ≤ 0.001). Renal MPO activity, but not serum NO level, in the treated group was similar to that in the control group (*P* > 0.05).

### 3.2. Gene Expression Study

#### 3.2.1. The Effect of D-lim on the Renal mRNA Expression of TNF-*α*, IL-6, and NF-*κ*B

**Figure 4:**
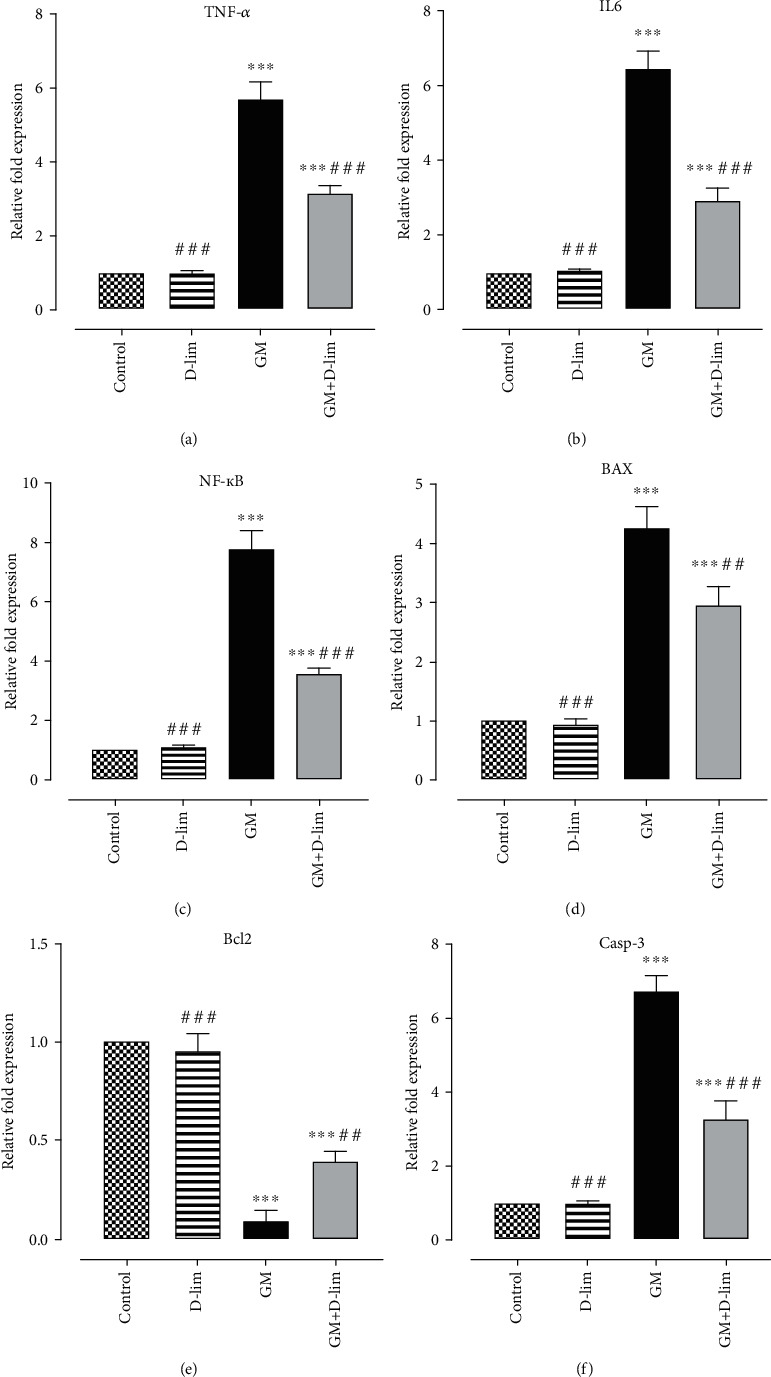
The effects of D-lim on the renal mRNA expression levels of inflammatory markers (a–c) and apoptotic indices (d–f) in rats with GM nephrotoxicity. Bars indicate mean ± SEM. One-way ANOVA followed by Tukey's post hoc test was used for comparison between groups. ^∗∗∗^*P* ≤ 0.001 against the control group. ^##^*P* ≤ 0.01 and ^###^*P* ≤ 0.001, against the GM group.

Gene expression analysis indicated that GM caused a significant (5.72-fold, 6.44-fold, and 7.78-fold, respectively) upregulation in the mRNA expression levels of TNF-*α*, IL-6, and NF-*κ*B in the GM group in comparison to the control group (*P* ≤ 0.001) (Figures 4(a)–4(c)). On the contrary, treatment with D-lim led to a notable downregulation in the mRNA expression levels of all target genes by 1.80-fold, 2.20-fold, and 2.17-fold, respectively (*P* ≤ 0.01) (Figures 4(a)–4(c)).

#### 3.2.2. The Effect of D-lim on the Renal mRNA Expression of Apoptotic Markers

Our results demonstrated that GM injection significantly upregulated the mRNA expression levels of the proapoptotic markers, Bax and Casp-3, by 4.25-fold and 6.70-fold, respectively (*P* ≤ 0.001). On the contrary, GM significantly suppressed the mRNA expression level of the antiapoptotic marker, Bcl2, by 10.76-fold (*P* ≤ 0.001). Inversely, D-lim significantly suppressed (1.43-fold and 2.07-fold, respectively) Bax and Casp-3 mRNA expression levels and upregulated Bcl2 mRNA expression (4.24-fold) in treated nephrotoxic rats compared to the GM group (*P* ≤ 0.01).

### 3.3. Histopathological Evaluation

**Figure 5:**
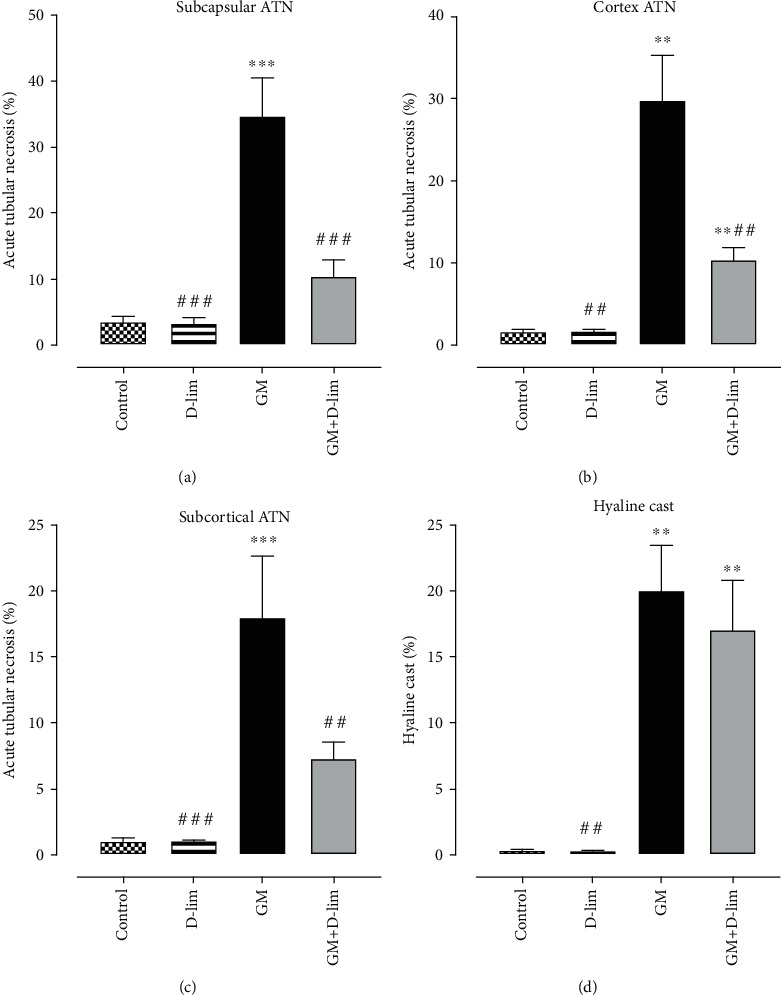
The effects of D-lim on GM-induced renal histopathological changes in rats intoxicated with GM. Bars indicate mean ± SEM. Data with normal distribution (subcapsular ATN and subcortical ATN) were compared between groups using one-way ANOVA followed by a post hoc LSD test. Nonnormal distribution data (cortex ATN and hyaline cast) were compared between groups using the Mann–Whitney *U* test. ^∗∗^*P* ≤ 0.01 and ^∗∗∗^*P* ≤ 0.001, against the control group. ^#^*P* < 0.05, ^##^*P* ≤ 0.01, and ^###^*P* ≤ 0.001, against the GM group.

**Figure 6:**
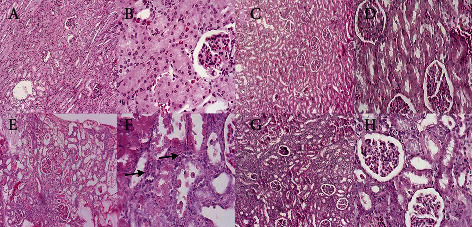
The effects of D-lim (100 mg/kg) on GM-induced kidney histopathological changes observed in the photomicrographs of renal sections stained with H&E. The control group (a, b) shows normal glomeruli and tubules. The D-lim group (c, d) indicates normal kidney architecture similar to the control group. The GM group (e, f) represents massive hyaline casts and tubulorrhexis (necrotic epithelial cells located in the centers of tubules). Additionally, infiltration of leukocytes to the parenchyma (arrows) was observed in the GM group (f). The treated group (g, h) displays amelioration of renal tissue with few casts, a few leukocytes, and desquamated tubules. (a, c, e, g) Low magnification (×100). (b, d, f, h) Higher magnification (×400).

The intensity of ATN and the level of hyaline cast formation in all groups are indicated in Figures 5 and 6. The severity of ATN in subcapsular, cortical, and subcortical regions and the level of hyaline cast formation in untreated nephrotoxic animals significantly amplified following GM injection compared to the control group (*P* ≤ 0.01). Oral treatment with D-lim led ATN severity in the investigated areas to decrease in the treated group compared to the GM group (*P* ≤ 0.01). However, the decrease in hyaline cast formation in the treated group following oral D-lim treatment was not statistically significant against the control group (*P* > 0.05). Prominent increase in leukocyte infiltration to the parenchyma was another histopathological finding in the GM group (Figure 6(f), arrows). Treatment with D-lim was able to reduce leukocyte infiltration in the treated animals (Figure 6(h)).

### 3.4. Immunohistochemical Analysis

**Figure 7:**
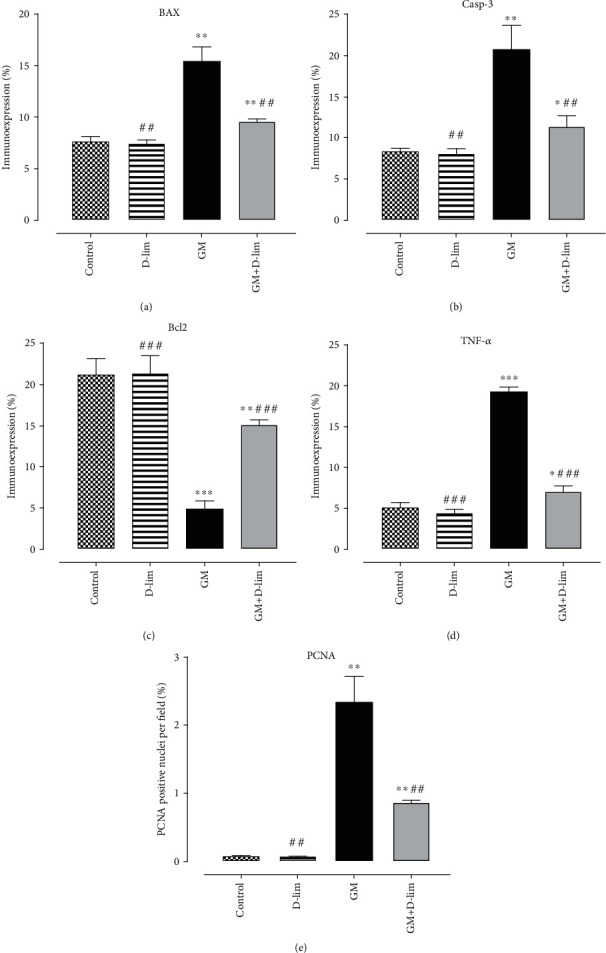
The effects of D-lim on immunoexpression of Bax (a), Casp-3 (b), Bcl2 (c), TNF-*α* (d), and PCNA (e) in the kidney of rats intoxicated with GM. Data with normal distribution (Bcl2 and TNF-*α*) were compared between groups using one-way ANOVA followed by a post hoc LSD test. Nonnormal distribution data (Bax, Casp-3, and PCNA) were compared between groups using the Mann–Whitney *U* test. ^∗^*P* < 0.05, ^∗∗^*P* ≤ 0.01, and ^∗∗∗^*P* ≤ 0.001, against the control group. ^##^*P* ≤ 0.01 and ^###^*P* ≤ 0.001, against the GM group.

**Figure 8:**
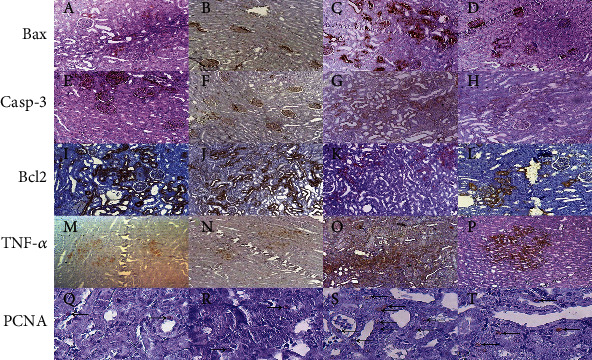
The effects of D-lim on immunoexpression of apoptotic biomarkers, TNF-*α*, and PCNA in the kidney of rats intoxicated with GM. IHC of Bax protein (a–d): a low population of only tubular cells expressing a proapoptotic marker was observed in the rat kidney related to the control (a) and D-lim (b) groups. Bax expression in both glomeruli and tubular cells enhanced in the GM group (b). Immunolabeling of an antibody in glomeruli but only in some tubules was observed in the GM+D-lim group (d). Anti-Casp-3 antibody expression (e–h): immunopositivity focused on some glomeruli and to a lesser extent tubules in the control (e) and D-lim (f) groups. A large population of epithelial cells immunoreacted to a Casp-3 antibody in the GM group (g). Decreased expression of Casp-3 in tubular cells was detected in the GM+D-lim group (h). Immunoexpression of Bcl2 (i–l): Bcl-2 has increased and has a strong cytoplasmic location in the control (i) and D-lim (j) groups but decreased in the GM group (k). Bcl2 immunoexpression considerably enhanced in the GM+D-lim group (l) compared with the GM group (k). Immunolabeling of TNF-*α* (m–p): low density of epithelial cells in the control (m) and D-lim (n) groups had cytoplasmic reaction. Diffuse and intensive immunostaining in the GM group (o) and focal positive reaction in the GM+D-lim group (p) were found in these IHC photomicrographs. Anti-PCNA protein (q–t): very few numbers of nuclei of epithelial cells have weak-to-moderate reaction to an antibody specified with arrows in the control (q) and D-lim (r) groups. The arrows indicate that larger numbers of nuclei are detected with a marker in the GM group (s) and the numbers of intermediate nuclei are stained in the GM+D-lim group (t).

The immunoexpression of Bax, Casp-3, Bcl2, TNF-*α*, and PCNA is depicted in Figures 7 and 8. Following GM injection for 12 days, the immunoexpression of Bax, Casp-3, PCNA, and TNF-*α* significantly (2.02-fold, 2.46-fold, 33.39-fold, and 3.77-fold, respectively) enhanced in the GM group compared to the control group (*P* ≤ 0.01). However, Bcl2 immunoreaction significantly (4.29-fold) decreased in GM-treated animals (*P* ≤ 0.001). 12 days of D-lim treatment significantly inhibited (1.61-fold, 1.82-fold, 2.71-fold, and 2.72-fold, respectively) the increase of Bax, Casp-3, TNF-*α*, and PCNA protein expressions as compared to the GM group (*P* < 0.05). Conversely, D-lim significantly promoted Bcl2 (3.05-fold) immunoexpression in the treated group (*P* ≤ 0.001).

## 4. Discussion

The present study was conducted for the first time to demonstrate the protective effects of oral D-lim on AKI following GM administration in rats. The acquired results revealed that AKI can be ameliorated by 12 days of oral D-lim treatment in GM-intoxicated animals. Oral treatment with D-lim for 12 days remarkably recovered most of the investigated parameters in GM-intoxicated animals.

Our results represented that GM markedly enhanced the serum levels of urea and Cr, which indicated GM-induced AKI. In many previous studies, a low glomerular filtration rate (GFR) following GM administration has been accounted for the marked elevations of urea and Cr [51, 52]. Moreover, it has been suggested that the tubular casts likely resulting from desquamating tubular epithelial cells and shedding brush borders may contribute to the decrease in GFR [53]. Accordingly, we observed a high level of hyaline cast formation in the lumen of tubules in GM-intoxicated animals. However, oral D-lim administration improved the serum levels of urea and Cr and ATN severity. These results were in compliance with previous studies indicating the renoprotective effects of D-lim [30, 54]. It has also been reported that other natural compounds with antioxidant activity such as gossypin [55], resveratrol [56], and ferulic acid [57] ameliorated renal histopathological changes and altered renal function after GM injection. Therefore, D-lim may increase GFR following the improvement of renal histopathological changes and finally reduce serum levels of urea and Cr.

GM is well known to promote ROS overproduction, which has a main role in the pathogenesis of GM-induced AKI. Consistent with different previous studies [4, 52, 55, 58], we observed that GM caused a significant elevation in MDA levels and a marked decrease in the levels of GSH and activities of antioxidant enzymes (SOD, GPX, and CAT) in the serum and kidney. Conversely, D-lim treatment was able to antagonize these alterations, but the increment in serum activities of SOD and CAT and decrease in serum MDA concentrations were not statistically significant. Our results are in accordance with former studies [21, 24–26, 59], which indicated the protective effects of D-lim against various oxidative stress conditions, and support the hypothesis that D-lim ameliorates GM-induced oxidative stress. These antioxidative protective effects of D-lim might be associated with its abilities to scavenge ROS [25, 60] and/or induce the gene expression of antioxidant enzymes.

It has been well established that NF-*κ*B and its downstream inflammatory cytokines play a main role in the pathogenesis of GM-induced AKI. In the present study, the upregulation of NF-*κ*B, IL-6, and TNF-*α* was observed following GM injection. Our results exhibited that D-lim significantly alleviated GM-induced inflammation via downregulating NF-*κ*B and subsequently suppressing the expression of IL-6 and TNF-*α* in nephrotoxic animals. These results corroborate previous studies implying anti-inflammatory effects attributed to D-lim and other natural antioxidants [28, 58, 61–63]. The mitogen-activated protein kinase (MAPK) pathway is known as an upstream effector involved in NF-*κ*B activation and then regulation of proinflammatory cytokine production [64, 65]. Interestingly, Chi et al. showed that D-lim anti-inflammatory effects are associated with the inactivation of both MAPK and NF-*κ*B pathways [61]. Taken together, the anti-inflammatory activity of D-lim observed in our study may be related to the inhibition of the MAPK pathway in addition to NF-*κ*B downregulation. D-lim effects on the MAPK pathway need to be further investigated in our animal model.

Our findings illustrated a marked increase in inflammatory cell infiltration in GM-intoxicated animals (Figure 6(f), arrows). Furthermore, we found a dramatic increase in renal MPO activity in untreated nephrotoxic animals, representing leukocyte infiltration. By contrast, D-lim markedly reduced leukocyte infiltration as affirmed by the reduction of renal MPO activity. The effects of D-lim on leukocyte infiltration and MPO activity may be related to the repression of inflammatory cytokines via downregulation of NF-*κ*B. These results represent further evidences to support anti-inflammatory effects of D-lim [66–68].

NO has a double-edged role and exhibits contradictory effects in the regulation of renal function. At low physiological concentration, NO is a vasodilator and also has a main role in the regulation tubular function [69]. However, NO at high levels generated by iNOS is associated with oxidative stress and has deleterious effects on the kidney [70, 71]. We and others [10, 58, 72] observed a high level of NO and severe renal damage in GM-injected animals. This increment in NO level has been attributed to GM-induced iNOS overexpression [10, 52]. D-lim administration could considerably reverse GM effects on NO level in treated animals. Our results came aligned with the study of Rehman et al. [30] who showed that D-lim reduced iNOS expression and NO level in a rodent model of doxorubicin nephrotoxicity. It can be speculated that D-lim effects on NO level may be associated with the suppression of iNOS expression and it needs to be furthered investigated in our rodent model.

Similar to the present study, different studies reported that GM nephrotoxicity disturbs the Bax/Bcl2 ratio through upregulation of Bax and downregulation of Bcl2, and it finally activates Casp-3 [73–75]. Conversely, D-lim treatment significantly inhibited the overexpression of proapoptotic proteins Bax and Casp-3 in the treated group compared to the GM group, which was consistent with the previous studies [25, 58, 73]. On the contrary, D-lim also could significantly increase the expression of Bcl2. These findings clearly revealed the antiapoptotic effects of D-lim against GM-induced AKI through the suppression of proapoptotic proteins and the upregulation of Bcl2. It has been demonstrated that p38MAPK mediated NF-*κ*B activation involved in GM-related apoptosis in the kidney [75]. NF-*κ*B proapoptotic effects have been attributed to the upregulation of proapoptotic proteins such as Casp-3 and P53 and the downregulation of Bcl2 [76]. Interestingly, Bai et al. showed that antiapoptotic effects of D-lim are associated with the inactivation of p38MAPK [25]. Moreover, similar to published data [54, 61], our results showed the decrease in oxidative stress by D-lim and its role in the inactivation of NF-*κ*B. Altogether, antiapoptotic effects of D-lim might be associated with its antioxidant properties and/or suppression of p38MAPK-mediated NF-*κ*B activation.

PCNA is well known to be involved in DNA repair and replication [15]. It has been previously reported that cisplatin as a nephrotoxic agent increased the expression of P53, P21, and PCNA suggesting cell cycle arrest and DNA repair [77]. Interestingly, Denamur et al. reported that GM in proximal cells caused a marked increase in the expression of P53 and P21, which can inhibit the cell cycle [18]. Additionally, similar to our study, other researches [19, 20] showed the overexpression of PCNA in the kidney of GM-intoxicated animals. Therefore, PCNA overexpression in the GM group may be associated with cell cycle arrest and the involvement of PCNA in DNA repair against GM-induced DNA damage [78]. By contrast, treatment with D-lim noticeably declined PCNA expression in comparison to untreated nephrotoxic animals, which was consistent with previous studies [19, 79]. One speculation about this effect is the amelioration of DNA damage by D-lim as previously reported by Bacanlı et al. [80].

## 5. Conclusions

In summary, the results of this study showed that D-lim could alleviate AKI following GM administration in rats. The protective effects of D-lim against GM-induced AKI may be associated with its antioxidant, anti-inflammatory, and antiapoptotic activities as well as downregulation of PCNA expression.

## Figures and Tables

**Table 1:** The sequence of utilized primers for QRT-PCR.

Gene	GeneBank Acc. no.	Primer position	Primer sequences (5′ → 3′)	Product length (bp)
Casp-3	NM_012922.2	Forward Reverse	GGACAGCAGTTACAAAATGGATTA CGGCAGGCCTGAATGATGAAG	393
Bax	NM_017059.2	Forward Reverse	CGTGGTTGCCCTCTTCTACTTT GATCAGCTCGGGCACTTTAGTG	73
Bcl-2	NM_016993.1	Forward Reverse	GATGACTTCTCTCGTCGCTA GTCATCCACAGAGCGATGTT	230
TNF-*α*	NM_012675.3	Forward Reverse	CCAGGAGAAAGTCAGCCTCCT TCATACCAGGGCTTGAGCTCA	87
IL-6	NM_012589.2	Forward Reverse	CGAAAGTCAACTCCATCTGCC GGCAACTGGCTGGAAGTCTCT	74
NF-*κ*B (p65)	NM_199267.2	Forward Reverse	AACACTGCCGAGCTCAAGAT CATCGGCTTGAGAAAAGGAG	163
GAPDH	NM_017008.4	Forward Reverse	ATGGAGAAGGCTGGGGCTCACCT AGCCCTTCCACGATGCCAAAGTTGT	209

## Data Availability

The datasets used and/or analyzed during the current study are available from the corresponding authors on reasonable request.

## References

[1] Raveh D., Kopyt M., Hite Y., Rudensky B., Sonnenblick M., Yinnon A. M. (2002). Risk factors for nephrotoxicity in elderly patients receiving once-daily aminoglycosides. *QJM*.

[2] Martinez-Salgado C., Lopez-Hernandez F. J., Lopez-Novoa J. M. (2007). Glomerular nephrotoxicity of aminoglycosides. *Toxicology and Applied Pharmacology*.

[3] Balakumar P., Chakkarwar V. A., Kumar V., Jain A., Reddy J., Singh M. (2008). Experimental models for nephropathy. *Journal of the Renin-Angiotensin-Aldosterone System*.

[4] Tavafi M., Ahmadvand H. (2011). Effect of rosmarinic acid on inhibition of gentamicin induced nephrotoxicity in rats. *Tissue and Cell*.

[5] Rodrigues F. A. P., Prata M. M. G., Oliveira I. C. M. (2014). Gingerol fraction from Zingiber officinale protects against gentamicin-induced nephrotoxicity. *Antimicrobial Agents and Chemotherapy*.

[6] Moreira M. A., Nascimento M. A., Bozzo T. A. (2014). Ascorbic acid reduces gentamicin-induced nephrotoxicity in rats through the control of reactive oxygen species. *Clinical Nutrition*.

[7] Randjelovic P., Veljkovic S., Stojiljkovic N., Sokolovic D., Ilic I. (2017). Gentamicin nephrotoxicity in animals: current knowledge and future perspectives. *EXCLI Journal*.

[8] Lopez-Novoa J. M., Quiros Y., Vicente L., Morales A. L., Lopez-Hernandez F. J. (2011). New insights into the mechanism of aminoglycoside nephrotoxicity: an integrative point of view. *Kidney International*.

[9] Vysakh A., Abhilash S., Kuriakose J., Midhun S. J., Jyothis M., Latha M. S. (2018). Protective effect of Rotula aquatica Lour against gentamicin induced oxidative stress and nephrotoxicity in Wistar rats. *Biomedicine & Pharmacotherapy*.

[10] Ozbek E., Cekmen M., Ilbey Y. O., Simsek A., Polat E. C., Somay A. (2009). Atorvastatin prevents gentamicin-induced renal damage in rats through the inhibition of p38-MAPK and NF-kB pathways. *Renal Failure*.

[11] Tugco V., Ozbek E., Tasci A. L. (2006). Selective nuclear factor *κ*-B inhibitors, pyrolidium dithiocarbamate and sulfasalazine, prevent the nephrotoxicity induced by gentamicin. *BJU International*.

[12] Morales A. L., Detaille D., Prieto M. (2010). Metformin prevents experimental gentamicin-induced nephropathy by a mitochondria-dependent pathway. *Kidney International*.

[13] Kalkan Y., Kapakin K. A. T., Kara A. (2012). Protective effect of Panax ginseng against serum biochemical changes and apoptosis in kidney of rats treated with gentamicin sulphate. *Journal of Molecular Histology*.

[14] Sepand M. R., Gharemani M. H., Razavi-Azarkhiavi K. (2016). Ellagic acid confers protection against gentamicin-induced oxidative damage, mitochondrial dysfunction and apoptosis-related nephrotoxicity. *The Journal of Pharmacy and Pharmacology*.

[15] Micol Tillhon O. C., Dutto I., Stivala L. A., Prosperi E. (2013). P21CDKN1A and DNA repair systems: recent findings and future perspectives. *New Research Directions in DNA Repair. vol 249-279*.

[16] Cazzalini O., Donà F., Savio M. (2010). p21^CDKN1A^ participates in base excision repair by regulating the activity of poly(ADP-ribose) polymerase-1. *DNA Repair*.

[17] Mocquet V., Lainé J. P., Ridel T., Yajin Z., Lee M. Y., Egly J. M. (2008). Sequential recruitment of the repair factors during NER: the role of XPG in initiating the resynthesis step. *The EMBO Journal*.

[18] Denamur S., Boland L., Beyaert M. (2016). Subcellular mechanisms involved in apoptosis induced by aminoglycoside antibiotics: insights on p53, proteasome and endoplasmic reticulum. *Toxicology and Applied Pharmacology*.

[19] Bledsoe G., Shen B., Yao Y.-Y. (2008). Role of tissue kallikrein in prevention and recovery of gentamicin-induced renal injury. *Toxicological Sciences*.

[20] Salama S. A., Arab H. H., Maghrabi I. A. (2018). Troxerutin down-regulates KIM-1, modulates p38 MAPK signaling, and enhances renal regenerative capacity in a rat model of gentamycin-induced acute kidney injury. *Food & Function*.

[21] Santiago J. V. A., Jayachitra J., Shenbagam M., Nalini N. (2012). Dietary d-limonene alleviates insulin resistance and oxidative stress-induced liver injury in high-fat diet and L-NAME-treated rats. *European Journal of Nutrition*.

[22] Rodríguez A., Shimada T., Cervera M. (2015). Resistance to pathogens in terpene down-regulated orange fruits inversely correlates with the accumulation of D-limonene in peel oil glands. *Plant Signal and Behavior*.

[23] Lu X. G., Zhan L. B., Feng B. A., Qu M. Y., Yu L. H., Xie J. H. (2004). Inhibition of growth and metastasis of human gastric cancer implanted in nude mice byd-limonene. *World Journal of Gastroenterology*.

[24] Yu L., Yan J., Sun Z. (2017). D-Limonene exhibits anti-inflammatory and antioxidant properties in an ulcerative colitis rat model via regulation of iNOS, COX-2, PGE2 and ERK signaling pathways. *Molecular Medicine Reports*.

[25] Bai J., Zheng Y., Wang G., Liu P. (2016). Protective effect of D-limonene against oxidative stress-induced cell damage in human lens epithelial cells via the p38 pathway. *Oxidative Medicine and Cellular Longevity*.

[26] Bacanlı M., Anlar H. G., Aydın S. (2017). d-limonene ameliorates diabetes and its complications in streptozotocin- induced diabetic rats. *Food and Chemical Toxicology*.

[27] Roberto D., Micucci P., Sebastian T., Graciela F., Anesini C. (2010). Antioxidant activity of limonene on normal murine lymphocytes: relation to H_2_O_2_ modulation and cell proliferation. *Basic & Clinical Pharmacology & Toxicology*.

[28] Del Toro-Arreola S., Flores-Torales E., Torres-Lozano C. (2005). Effect of d-limonene on immune response in BALB/c mice with lymphoma. *International Immunopharmacology*.

[29] d'Alessio P., Mirshahi M., Bisson J.-F., Bene M. (2014). Skin repair properties of d-Limonene and perillyl alcohol in murine models. *Antiinflammatory and Antiallergy Agents in Medicinal Chemistry*.

[30] Rehman M. U., Tahir M., Khan A. Q. (2014). D-limonene suppresses doxorubicin-induced oxidative stress and inflammation via repression of COX-2, iNOS, and NF*κ*B in kidneys of Wistar rats. *Experimental Biology and Medicine*.

[31] Sun J. (2007). D-Limonene: safety and clinical applications. *Alternative Medicine Review*.

[32] Murali R., Saravanan R. (2012). Antidiabetic effect of d-limonene, a monoterpene in streptozotocin-induced diabetic rats. *Biomedicine & Preventive Nutrition*.

[33] Kang C., Lee H., Hah D. Y. (2013). Protective effects of Houttuynia cordata Thunb. on gentamicin-induced oxidative stress and nephrotoxicity in rats. *Toxicological Research*.

[34] Ahmadvand H., Ghasemi Dehnoo M., Dehghani A., Bagheri S., Cheraghi R. A. (2013). Serum paraoxonase 1 status and its association with atherogenic indexes in gentamicin-induced nephrotoxicity in rats treated with coenzyme Q10. *Renal Failure*.

[35] Lee I.-C., Kim S.-H., Lee S.-M. (2012). Melatonin attenuates gentamicin-induced nephrotoxicity and oxidative stress in rats. *Archives of Toxicology*.

[36] Spector A. C., Grill H. J. (1992). Salt taste discrimination after bilateral section of the chorda tympani or glossopharyngeal nerves. *American Journal of Physiology*.

[37] Bouairi E., Neff R., Evans C., Gold A., Andresen M. C., Mendelowitz D. (2004). Respiratory sinus arrhythmia in freely moving and anesthetized rats. *Journal of Applied Physiology*.

[38] Ohkawa H., Ohishi N., Yagi K. (1979). Assay for lipid peroxides in animal tissues by thiobarbituric acid reaction. *Analaytical Biochemistry*.

[39] Ahmadvand H., Babaeenezhad E., Nayeri H., Zarei Nezhad Z. (2019). Selenium effects on antioxidant and inflammatory indices in renal ischemia-reperfusion injury in rats. *Journal of Renal Injury Prevention*.

[40] Ahmadvand H., Yalameha B., Adibhesami G. (2019). The protective role of gallic acid pretreatment on renal ischemia-reperfusion injury in rats. *Reports of Biochemistry and Molecular Biology*.

[41] Ellman G. L. (1959). Tissue sulfhydryl groups. *Archives of Biochemistry and Biophysics*.

[42] Giustarini D., Rossi R., Milzani A., Dalle-Donne I. (2008). Nitrite and nitrate measurement by Griess reagent in human plasma: evaluation of interferences and standardization. *Methods in Enzymology*.

[43] Sinha A. K. (1972). Colorimetric assay of catalase. *Analaytical Biochemistry*.

[44] Rotruck J. T., Pope A. L., Ganther H. E., Swanson A. B., Hafeman D. G., Hoekstra W. G. (1973). Selenium: biochemical role as a component of glutathione peroxidase. *Science*.

[45] Kuthan H., Haussmann H. J., Werringloer J. (1986). A spectrophotometric assay for superoxide dismutase activities in crude tissue fractions. *Biochemistry Journal*.

[46] Mullane K. (1989). Neutrophil-platelet interactions and post-ischemic myocardial injury. *Progress in Clinical and Biological Research*.

[47] Jamor P., Ahmadvand H., Ashoory H., Babaeenezhad E. (2019). Effect of alpha-lipoic acid on antioxidant gene expression and kidney injury in alloxan-induced diabetic rats. *Journal of Nephropathology*.

[48] Livak K. J., Schmittgen T. D. (2001). Analysis of relative gene expression data using real-time quantitative PCR and the 2^−*ΔΔ* _C_^_T_ method. *Methods*.

[49] Soares T. J., Costa R. S., Volpini R. A., Da Silva C. G., Coimbra T. M. (2002). Long-term evolution of the acute tubular necrosis (ATN) induced by glycerol: role of myofibroblasts and macrophages. *International Journal of Experimental Pathology*.

[50] Al Seyedan A., Dezfoulian O., Alirezaei M. (2020). Satureja khuzistanica Jamzad essential oil prevents doxorubicin-induced apoptosis via extrinsic and intrinsic mitochondrial pathways. *Research in Pharmaceutical Sciences*.

[51] Laurent G., Kishore B. K., Tulkens P. M. (1990). Aminoglycoside-induced renal phospholipidosis and nephrotoxicity. *Biochemical Pharmacology*.

[52] Abd-Elhamid T. H., Elgamal D. A., Ali S. S. (2018). Reno-protective effects of ursodeoxycholic acid against gentamicin-induced nephrotoxicity through modulation of NF-*κ*B, eNOS and caspase-3 expressions. *Cell and Tissue Research*.

[53] Hosaka E. M., Santos O. F. P., Seguro A. C., Vattimo M. F. F. (2004). Effect of cyclooxygenase inhibitors on gentamicin-induced nephrotoxicity in rats. *Brazilian Journal of Medical and Biological Research*.

[54] Murali R., Karthikeyan A., Saravanan R. (2013). Protective effects of d-limonene on lipid peroxidation and antioxidant enzymes in streptozotocin-induced diabetic rats. *Basic and Clinical Pharmacology and Toxicology*.

[55] Abdelrahman R. S. (2017). Protective effect of apocynin against gentamicin-induced nephrotoxicity in rats. *Human & Experimental Toxicology*.

[56] Beshay O. N., Ewees M. G., Abdel-Bakky M. S., Hafez S. M. N. A., Abdelrehim A. B., Bayoumi A. M. A. (2020). Resveratrol reduces gentamicin-induced EMT in the kidney via inhibition of reactive oxygen species and involving TGF-*β*/Smad pathway. *Life Sciences*.

[57] Erseçkin V., Mert H., İrak K., Yildirim S., Mert N. (2020). Nephroprotective effect of ferulic acid on gentamicin-induced nephrotoxicity in female rats. *Drug and Chemical Toxicology*.

[58] Adil M., Kandhare A. D., Dalvi G. (2016). Ameliorative effect of berberine against gentamicin-induced nephrotoxicity in rats via attenuation of oxidative stress, inflammation, apoptosis and mitochondrial dysfunction. *Renal Failure*.

[59] Wang X., Li G., Shen W. (2018). Protective effects of D-limonene against transient cerebral ischemia in stroke-prone spontaneously hypertensive rats. *Experimental and Therapeutic Medicine*.

[60] Chaudhary S. C., Siddiqui M. S., Athar M., Alam M. S. (2012). D-Limonene modulates inflammation, oxidative stress and Ras-ERK pathway to inhibit murine skin tumorigenesis. *Human Experimental Toxicology*.

[61] Chi G., Wei M., Xie X., Soromou L. W., Liu F., Zhao S. (2013). Suppression of MAPK and NF-*κ*B pathways by limonene contributes to attenuation of lipopolysaccharide-induced inflammatory responses in acute lung injury. *Inflammation*.

[62] Ahmad S. B., Rehman M. U., Fatima B. (2018). Antifibrotic effects of D-limonene (5(1-methyl-4-[1-methylethenyl]) cyclohexane) in CCl4-induced liver toxicity in Wistar rats. *Environmental Toxicology*.

[63] Mahmoud Y. I. (2017). Kiwi fruit (Actinidia deliciosa) ameliorates gentamicin-induced nephrotoxicity in albino mice via the activation of Nrf2 and the inhibition of NF-*κ*B (Kiwi & gentamicin-induced nephrotoxicity). *Biomedicine and Pharmacotherapy*.

[64] Polat A., Parlakpinar H., TasdemirA S. (2006). Protective role of aminoguanidine on gentamicin-induced acute renal failure in rats. *Acta Histochemica*.

[65] Lee K. E., Kim E. Y., Kim C. S. (2013). Macrophage-stimulating protein attenuates gentamicin-induced inflammation and apoptosis in human renal proximal tubular epithelial cells. *Biochemical and Biophysical Research Communications*.

[66] Chen X.-L., Zhang Q., Zhao R., Medford R. M. (2004). Superoxide, H2O2, and iron are required for TNF-*α*-induced MCP-1 gene expression in endothelial cells: role of Rac1 and NADPH oxidase. *American Journal of Physiology-Heart and Circulatory Physiology*.

[67] Segerer S., Nelson P. J., Schlondorff D. (2000). Chemokines, chemokine receptors, and renal disease: from basic science to pathophysiologic and therapeutic studies. *Journal of the American Society of Nephrology*.

[68] Spiecker M., Darius H., Kaboth K., Hübner F., Liao J. K. (1998). Differential regulation of endothelial cell adhesion molecule expression by nitric oxide donors and antioxidants. *Journal of Leukocyte Biology*.

[69] Radermacher J., Klanke B., Schurek H. J., Stolte H. F., Frölich J. C. (1992). Importance of NO/EDRF for glomerular and tubular function: studies in the isolated perfused rat kidney. *Kidney International*.

[70] Christo J. S., Rodrigues A. M., Mouro M. G. (2011). Nitric oxide (NO) is associated with gentamicin (GENTA) nephrotoxicity and the renal function recovery after suspension of GENTA treatment in rats. *Nitric Oxide*.

[71] Araujo M., Welch W. J. (2006). Oxidative stress and nitric oxide in kidney function. *Current Opinion in Nephrology and Hypertension*.

[72] El-Kashef D. H., El-Kenawi A. E., Sudek G. M., Salem H. A. (2015). Protective effect of allicin against gentamicin-induced nephrotoxicity in rats. *International Immunopharmacology*.

[73] El Gamal A. A., AlSaid M. S., Raish M. (2014). Beetroot (Beta vulgaris L.) extract ameliorates gentamicin-induced nephrotoxicity associated oxidative stress, inflammation, and apoptosis in rodent model. *Mediators of Inflammation*.

[74] Shin H. S., Yu M., Kim M., Choi H. S., Kang D. H. (2014). Renoprotective effect of red ginseng in gentamicin-induced acute kidney injury. *Laboratory Investigation*.

[75] Gong X., Celsi G., Carlsson K., Norgren S. (2012). Protective effects ofN-Acetylcysteine amide (NACA) on gentamicin-induced apoptosis in LLC-PK1 cells. *Renal Failure*.

[76] Chen Y. C., Chen C. H., Hsu Y. H. (2011). Leptin reduces gentamicin-induced apoptosis in rat renal tubular cells via the PI3K-Akt signaling pathway. *European Journal of Pharmacology*.

[77] Potocnjak I., Domitrovic R. (2016). Carvacrol attenuates acute kidney injury induced by cisplatin through suppression of ERK and PI3K/Akt activation. *Food and Chemical Toxicology*.

[78] Choi K. H., Kim T. I., Chong D. L., Lee H. Y., Han D. S. (2000). Gentamicin induced apoptosis of renal tubular epithelial LLC-PK1 cells. *The Korean Journal of Internal Medicine*.

[79] Biro A., Vaknine H., Cohen-Armon M. (2016). The effect of poly (ADP-ribose) polymerase inhibition on aminoglycoside-induced acute tubular necrosis in rats. *Clinal Nephrology*.

[80] Bacanlı M., Başaran A. A., Başaran N. (2015). The antioxidant and antigenotoxic properties of citrus phenolics limonene and naringin. *Food and Chemical Toxicology*.

